# Access to primary care for persons with spinal cord injuries in the greater Gaborone area, Botswana

**DOI:** 10.4102/ajod.v8i0.539

**Published:** 2019-09-23

**Authors:** Thato M.M. Paulus-Mokgachane, Surona J. Visagie, Gubela Mji

**Affiliations:** 1 Spinal cord injury rehabilitation Unit, Princess Marina Hospital, University of Botswana, Gaborone, Botswana; 2 Centre for Rehabilitation Studies, Stellenbosch University, Cape Town, South Africa

**Keywords:** spinal cord injury, primary care, Botswana, access, available, affordable, accessible, acceptable, adequate

## Abstract

**Background:**

People with spinal cord injury (SCI) often have great need for healthcare services, but they report access challenges. Primary care access to people with SCI has not been explored in Botswana.

**Objective:**

This study aimed to identify barriers and facilitators that users with spinal cord injuries experience in accessing primary care services in the greater Gaborone area, Botswana.

**Methods:**

A quantitative, cross-sectional, observational study was conducted. Data were collected with a structured questionnaire from 57 participants with traumatic and non-traumatic SCI. Descriptive and inferential analysis was performed.

**Results:**

The male to female ratio was 2.8:1. The mean age of participants was 40 years (standard deviation 9.59). Road traffic crashes caused 85% of the injuries. Most participants visited primary care facilities between 2 and 10 times in the 6 months before the study. Participants were satisfied with the services (63%) and felt that facilities were clean (95%) and well maintained (73.5%). Preferential treatment, respect, short waiting times and convenient hours facilitated satisfaction with services. Availability was hampered by insufficient provider knowledge on SCI as indicated by 71.9% of participants, and shortage of consumables (80.7%). Structural challenges (42.1% could not enter the facility by themselves and 56.5% could not use the bathroom) and lack of height-adjustable examining couches (66.7%) impeded accessibility. Cost was incurred when participants (64.9%) utilised private health services where public services failed to address their needs.

**Conclusion:**

Primary care services were mostly affordable and adequate. Availability, acceptability and accessibility aspects created barriers.

## Introduction

Persons with disabilities can experience excellent health, but because of the nature of the impairment, co-morbidities and secondary complications, they often require more access to healthcare than persons without disabilities (Shakespeare 2014). According to a systematic review by Bright and Kuper (2018), studies have shown that persons with disabilities experience greater barriers in accessing healthcare than persons without disabilities. The barriers include geographic and transport difficulties, financial challenges, staff attitudes, inaccessible buildings, a lack of equipment, communication barriers and lack of skills and knowledge amongst service providers (Baart & Taaka 2017; Bright & Kuper 2018). Persons with disabilities are also more likely to be dissatisfied with healthcare services than their non-disabled peers (Gulley & Altman 2008; Parish & Huh 2006).

Spinal cord injury (SCI) is often associated with poorer health outcomes because of secondary complications and challenges in accessing healthcare (Amatachaya et al. 2011; Chamberlain et al. 2015; Hitzig et al. 2008; Löfvenmark et al. 2017b; Øderud 2014). Common secondary complications after SCI include urinary tract infections, bowel problems, respiratory infections, autonomic dysreflexia, pressure ulcers, musculoskeletal and/or neuropathic pain, fractures and depression (Amatachaya et al. 2011; Hitzig et al. 2008; Löfvenmark et al. 2017b; Øderud 2014). Persons with SCI also remain at risk for health conditions seen in general populations such as cardiovascular diseases (Chamberlain et al. 2015; Hitzig et al. 2008). Thus, persons with SCI might need to utilise healthcare services more than persons without SCI.

Healthcare access for persons with SCI, as for their peers, starts with access to primary care. The role of primary care is to ‘bring promotion and prevention, cure and care together in a safe, effective and socially productive way at the interface between the population and the health system’ (WHO 2008:41). Primary care service delivery in Africa is hampered by inequity, ineffectiveness, poor coverage and access, lack of financial and human resources, poor governance and lack of monitoring and evaluation (Onokerhoraye 2016).

**FIGURE 1:**
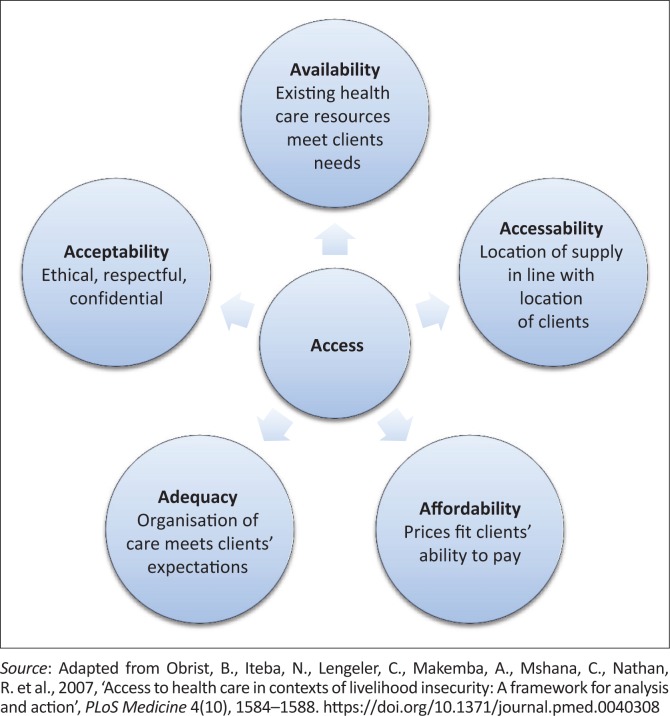
Healthcare access framework.

According to the ACCESS framework (Obrist et al. 2007), healthcare access and use is dependent on five dimensions (Figure 1).

*Availability of care:* Availability refers to an adequate supply of facilities, services, providers, consumables and drugs. It also infers that providers have adequate knowledge and skills (Obrist et al. 2007). Studies from North America report that primary care facilities and services were available to persons with SCI (Goodridge et al. 2015; Stillman et al. 2014, 2017). However, the number of providers was not always sufficient, with resultant long waiting times (Goodridge et al. 2015). Service providers’ knowledge and skills were also deemed insufficient to deal with the specific needs of someone with SCI and to understand the impact of SCI on overall health (Goodridge et al. 2015; Stillman et al. 2014, 2017).

African studies focusing on primary care access for persons with SCI specifically could not be identified. Studies that focused on persons with diverse disabilities showed challenges with service availability that included a lack of services and facilities (Eide et al. 2015; Mulumba et al. 2014), insufficient drugs (Eide et al. 2015; Mulumba et al. 2014; Van Rooy et al. 2012; Vergunst et al. 2015), insufficient equipment (Eide et al. 2015; Vergunst et al. 2015), lack of staff (Mlenzana & Mwansa 2012; Mulumba et al. 2014; Vergunst et al. 2015), lack of skills (Mlenzana et al. 2013; Mulumba et al. 2014; Van Rooy et al. 2012) and long waiting times (Cawood & Visagie 2015; Van Rooy et al. 2011; Vergunst et al. 2015).

*Accessibility of care:* An accessible service is within easy reach of clients in terms of distance, transport and travel time. It is also not hampered by architectural barriers at the facility and poor access to equipment, diagnostic and treatment services (Goodridge et al. 2015). The majority (73.8%) of American wheelchair users (*n* = 432) in a study by Stillman et al. (2017) experienced physical access challenges at primary care facilities; as did the majority (99.1%) of a group with SCI (*n* = 108) (Stillman et al. 2014). Doors, hallways, bathrooms and examination rooms created barriers (Stillman et al. 2014, 2017). A lack of height-adjustable beds, combined with a lack of transfer equipment, led to many participants (69.7% – Stillman et al. 2017; 85.2% – Stillman et al. 2014) being examined in their wheelchairs.

In Africa, people frequently walk, often over considerable distances and inhospitable terrain to access healthcare. Therefore, mobility impairments together with a lack of mobility assistive devices and poor access to public transport because of unavailability, inaccessibility and high cost can create barriers to healthcare access for persons with disabilities (Cawood & Visagie 2015; Löfvenmark et al. 2017b; Maart & Jelsma 2013; Mulumba et al. 2014; Munthali et al. 2019; Van Rooy et al. 2012; Vergunst et al. 2015). At facilities, further physical barriers are encountered (Vergunst et al. 2015), which include inaccessible bathrooms and inaccessible entrances (Van Rooy et al. 2011).

*Affordability of care:* Affordability is impacted by direct and indirect costs including loss of income, transport costs, time loss and cost of drugs, consumables and consultations (Obrist et al. 2007). Van Rooy et al. (2012), Munthali et al. (2019) and Maart and Jelsma (2013) identified costs of accessing healthcare to be a significant factor that limits healthcare access of persons with disabilities in Namibia, Malawi and South Africa.

*Adequacy of care:* Adequacy refers to the organisation of care in terms of the facilities’ hours as well as to cleanliness and maintenance of the facility and equipment. Van Rooy et al. (2011) identify challenges with appointment systems, facility maintenance and cleanliness. Scheffler, Visagie and Schneider (2015) have shown how an appointment for a specific time, triage and extended service hours improved adequacy of services at an urban primary care facility in South Africa.

*Acceptability of care:* An acceptable service is ethically sound, and appropriate to gender, life cycle and cultural needs of clients. Confidentiality and privacy are ensured. Participants in the qualitative study by Goodridge et al. (2015) felt that in order to protect their health interests, they had to be vigilant and proactive and not unquestioningly accept the opinion of care providers.

Van Rooy et al. (2012) describe nurses to be rude and demonstrate a failure of clients with disabilities to fully utilise the available healthcare services because of staff attitude. Other African studies also found attitudinal barriers including a lack of compassion, patience, courtesy and respect (Mlenzana & Mwansa 2012; Mulumba et al. 2014; Munthali et al. 2019). Furthermore, Mlenzana and Mwansa (2012), Munthali et al. (2019) and Mulumba et al. (2014) identified communication challenges in Zambian, Malawian and Ugandan studies, respectively.

It can thus be concluded that healthcare access to persons with SCI in developed countries was hampered by challenges in all five access dimensions. Similarly, access to primary care for persons with disabilities in Africa was hampered in all five dimensions.

Löfvenmark and colleagues provide valuable information on the epidemiology of and outcomes after SCI, as well as on the experience of living with SCI in Botswana (Löfvenmark et al. 2015, 2016, 2017a, 2017b). However, there is a paucity of studies from Africa and Botswana that focus on primary care access for persons with SCI. The challenges and enablers that persons with SCI face when accessing primary care, a service that is a fundamental human right, remain unexplored. Therefore, this study with the aim to identify barriers and facilitators that users with SCI experience in accessing primary care services in the greater Gaborone city area, Botswana, evolved.

### Study setting

Botswana has a population of 2 024 904 inhabitants of which 231 592 reside in the capital city of Gaborone (Statistics Botswana 2014). According to 2011 population and housing census, there are 59 103 (2.92%) persons with disabilities in Botswana (Statistics Botswana 2014). Amongst these, 11.7% reported impairments of legs, arms (6.3%) and/or an inability to use the body (2.5%) (Hlalele et al. 2015).

Gaborone has a disability prevalence of 1.5%, which is below the national prevalence (Hlalele et al. 2015). Spinal cord injury prevalence figures could not be found for either the capital city or the entire country. Löfvenmark et al. (2015) found the incidence of traumatic SCI to be 13 per million per year at the only SCI rehabilitation unit, Spinalis Botswana (Spinalis), in the country.

There is a single public hospital, Princess Marina Hospital (PMH), in the city of Gaborone, which serves as a referral facility for the southern part of the country. Greater Gaborone also has two district hospitals, two private hospitals and a number of private clinics. Princess Marina Hospital houses the only SCI rehabilitation unit in the country. Established in partnership between the government of Botswana and a Swedish nongovernmental organisation (NGO) ‘Spinalis foundation’, the unit is called Botswana Spinalis SCI rehabilitation unit. Care and rehabilitation for persons with traumatic SCI are provided in a 12-bed ward in this unit. Persons with non-traumatic SCI are treated in other departments of PMH and at district hospitals.

Following discharge from hospital, both persons with traumatic and non-traumatic SCI are expected to source primary care and SCI-related consumables from their local primary care facilities. In Botswana, primary care is provided through health posts, clinics and district hospitals for a consultation fee of 5 Pula ($0.50). These facilities are managed by district health management teams. There are 74 public clinics and health posts in the study area.

## Methods

A quantitative, cross-sectional, observational survey was performed. The study population consisted of persons with SCI residing in the greater Gaborone city area. Persons older than 21 years, with a complete or incomplete, traumatic or non-traumatic SCI, were included in the study. People with co-morbidities like mental impairments or brain trauma were excluded. Neither the precise number of people nor names and contact details were on record for this population.

Since the establishment of Spinalis in 2010, a database of clients with traumatic SCI has been kept. At the time of the study, the database contained 197 names of people with traumatic SCI from the entire country, of whom 60 resided in the study setting. A complete database for persons with non-traumatic SCI could not be found. The databases of the orthopaedics department and the spine clinic at PMH were accessed to identify people with non-traumatic SCI living in the study setting. Six persons were identified.

Thus, a possible 66 participants were identified. The contact details of nine persons were incorrect. The rest (57; 51 with traumatic SCI and 6 with non-traumatic SCI) participated in the study.

A self-developed questionnaire, based on the ACCESS framework (Obrist et al. 2007), was used for data collection. The questionnaire was developed in English and translated into Setswana, with subsequent back translation to English.

Data were collected through verbal administration at a venue of participants’ choice by the first author. After the data were checked for errors, it was entered onto an Excel spreadsheet. Data were mainly categorical (nominal or ordinal) in nature. Descriptive analysis was performed, and prevalence ratios calculated to determine if certain variables such as gender, type of facility visited or level of injury impacted satisfaction with services and availability of services (Morroni & Myer 2007).

## Results

The male to female ratio amongst study participants was 2.8:1 with 73.7% (42) being men and 26.3% (15) women. The mean age of participants at the time of data collection was 40 (standard deviation [s.d.] 9.59), ranging from 22 to 64. On average, the years since the injury were 4 (s.d. 12), ranging from 2 to 5 years. The most common cause of SCI amongst the participants was road traffic crashes (RTCs) (48; 85%). Other causes such as violence (3; 5%), tuberculosis (3; 5%) and compressive myelopathy (3; 5%) were rare. Almost the same number of participants had paraplegia (28; 49.1%) and tetraplegia (29; 50.9%).

**FIGURE 2:**
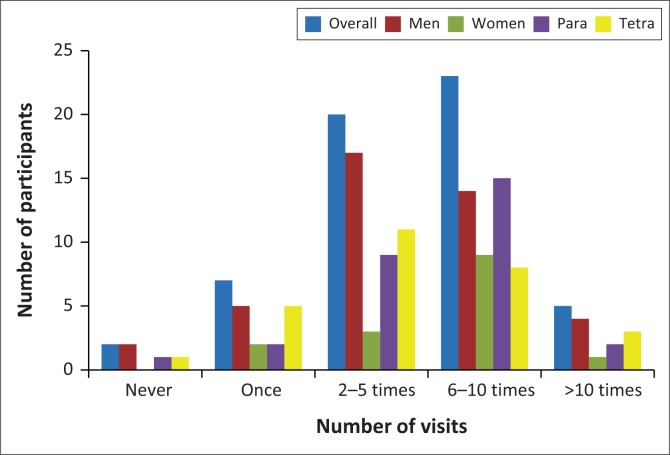
Frequency of primary care visits in the 6 months before the study (*n* = 57).

Figure 2 shows that most participants (43; 75%) visited primary care facilities between 2 and 10 times in the 6 month period before the study.

The most common reason for visiting the health facilities was SCI-related complications (22; 38.6%) followed by SCI-related consumables (18; 31.6%) and minor ailments (17; 29.8%). A higher percentage of women (9/15; 60%) visited a facility for minor ailments than men (8/42; 19%).

### Satisfaction with primary care

Thirty-six participants (63.2%) were always or mostly satisfied with the care they received, while 21(36.8%) were hardly or never satisfied. Prevalence ratios showed little difference between satisfaction with services and gender (0.94), level of injury (1.6) and type of facility visited (1.47).

### Availability of primary care

The majority of participants (41; 71.9%) accessed a clinic for primary care, while 11 (19.3%) accessed a hospital and five (8.8%) accessed a health post. Prevalence ratios showed that those who accessed a hospital felt that services were 7.5 times more available than those that access clinics or health posts.

Table 1 shows that the prescribed medication was always available for 45.6% (26) of participants and sometimes for a further 36.8% (21). This trend was observed for all three types of delivery points with somewhat lower availability at hospitals (40%) than clinics (45%) and the highest availability at health posts (55%). Consumables were less often available. Prevalence ratios show no difference between availability of medicine (0.52) and SCI consumables (1.3) between the different facilities.

**TABLE 1:** Availability of medication and consumables (*n* = 57).

Availability	Variable	Never or hardly ever	%	Sometimes	%	Always	%
Prescribed medication	Clinic	7	12	24	42.1	26	45.6
	Health post	15	27	10	17.6	32	55
	Hospital	23	40.4	11	19.3	23	40.4
	Overall	10	17.6	21	36.8	26	45.6
Consumables	Clinic	29	50.8	15	26.4	13	22.8
	Health post	35	60.4	11	19.3	11	19.3
	Hospital	25	45	27	46	5	9
	Overall	33	57.9	13	22.8	11	19.3

While 71.9% (41) of participants thought that the number of staff was excellent or good, they perceived challenges with regard to staff’s knowledge on SCI-related problems, with 71.9% (41) scoring this aspect as poor or very poor and none scoring it as excellent (Table 2). Staff at hospitals was 2.1 times more likely to have sufficient knowledge on SCI and hospitals were 4.2 more likely to have adequate staff numbers than the other facilities.

**TABLE 2:** Availability and knowledge of staff (*n* = 57).

Variable	Very poor or poor	%	Good	%	Excellent	%
**Availability of staff**	16	28	17	29.8	24	42.1
**Staff knowledge on spinal cord injury**	41	71.9	16	28.1	0	0

### Accessibility of primary care

**FIGURE 3:**
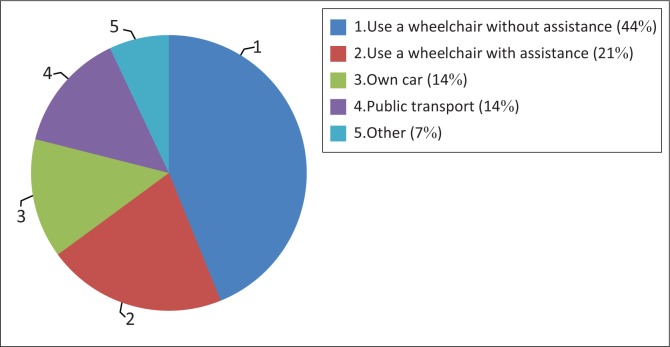
Mode of travel to the healthcare facility (*n* = 57).

While 84.2% (48) of participants stayed less than 5 km from their primary care facility, 52.6% (30) could not get to the facility easily. Figure 3 shows that most participants (37; 65%) used their wheelchairs with (12; 21%) or without assistance (25; 44%) to get to the healthcare facilities.

Twenty-four (42.1%) participants could not enter the facility by themselves. The reasons for this included the absence of a ramp (1), too steep a ramp (7), sandy or rough terrain outside (5), narrow door (4), inability to open the door (4), a door mat (1) and other not specified (2). Eleven (19.3%) participants have never attempted to use the bathroom at the healthcare facility. Of the 46 (80.7%) who did want to use the bathroom, the majority (26/46; 56.2%) were unable to access the toilet and use hand washing (22/46; 47.8%) and drying facilities (26/46; 56.2%). None of the participants had access to an emergency call button in the bathroom.

### Affordability of primary care

Most participants (48; 84.2%) were not required to pay for healthcare. Six of the participants paid more than 100 Pula ($10), two paid between 10 and 100 Pula ($1–$10), while one paid 5 Pula (< $1) (the amount charged by the government for consultations at the time of the study). However, 64.9% (37) of the participants incurred costs through having to access private services such as a doctor, medication or consumables when these were not available through the public service. Most (40; 70.2%) of the participants did not spend money to reach the primary care facilities. Four (7%) participants, however, spent more than 300 Pula ($30) to access the primary care facilities. The majority (41; 71.9%) of the participants held the view that accessing primary care services was not expensive.

### Acceptability of primary care

Table 3 shows that 59.6% (34) of the participants used facilities that had an appointment system of which 11 (19.3%) were given appointments for a specific time. The majority of participants waited less than 30 min for consultations (26; 46%) and at the dispensary (48; 84%).

**TABLE 3:** Service acceptability (*n* = 57).

Variable	Yes	%
Appointment system	34	59.6
Date and time	11	19.3
Only date	23	40.4
Assessed in wheelchair	37	64.9
Height-adjustable bed	19	33.3
Refused care a primary care facility	4	7
Referred to another facility	42	73.7
Transport offered with referral	8	14.0
Preferential treatment	42	73.7
Treated with dignity	51	89.5
Experience staff attitudes as positive	35	61.4

Most facilities (38; 66.7%) did not have height-adjustable beds; consequently, 37 participants (64.9%) were assessed in their wheelchairs. Forty-two (73.7%) participants had been referred to another facility and amongst those referred, 34 (59.6%) had no transport offered to them.

Most participants (42; 73.7%) stated that they received preferential treatment and that they were treated with dignity (51; 89.5%). With regard to staff attitude towards them, 35 (61.4%) of the participants reported that it was positive.

### Adequacy of primary care services

**FIGURE 4:**
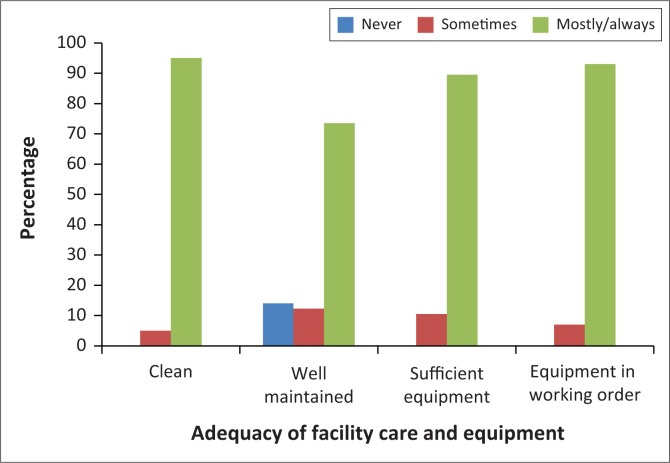
Participants’ opinions on aspects related to the adequacy of primary care (*n* = 57).

The majority of participants (51; 89.5%) were of the opinion that the facilities were open at hours convenient for them. They also indicated high levels of satisfaction with cleanliness, facility maintenance and the availability of functioning equipment, as shown in Figure 4.

## Discussion

### Demographic information

The higher ratio of men to women is consistent with worldwide trends in SCI (Singh et al. 2014). The percentage of men is also similar to that found by Löfvenmark et al. (2015) in a previous study conducted in Botswana. The finding that participants were on average younger than 40 years old is also consistent with international and national data on SCI (Löfvenmark et al. 2015; Singh et al. 2014). The low average time since the injury could indicate poor long-term survival of people with SCI in Botswana. However, further study is necessary to come to any definite conclusion on this.

### Medical information

The percentage of participants who were injured in RTCs is higher than the 68% found by Löfvenmark et al. (2015) in Botswana and the 41% described by Rahimi-Movaghar et al. (2013) in developing countries. This might be because of sampling bias in the current study or it might indicate an unduly high number of RTCs in Botswana. Other traumatic causes as well as medical causes were rare. However, one cannot conclude that traumatic SCI occurred at higher frequency than non-traumatic SCI in the study setting. A database for persons with traumatic SCI was readily available, while there was none for persons with non-traumatic injuries.

The finding that similar numbers of participants had paraplegia (28; 49.1%) and tetraplegia (29; 50.9%) is inconsistent with findings from Löfvenmark et al. (2015), where tetraplegia was said to be more common than paraplegia. This difference could be because of a higher mortality rate of people with tetraplegia when compared to those with paraplegia as noted by Löfvenmark et al. (2015) and Øderud (2014). Further studies are, however, required to investigate this hypothesis.

### Primary care visits

The results demonstrated a big need for healthcare services amongst people with SCI which is consistent with literature findings (Amatachaya et al. 2011; Chamberlain et al. 2015; Hitzig et al. 2009; Øderud 2014). The finding that women visited the facilities more often than men is in accordance with findings on primary care use of general populations (Mash et al. 2012). The majority of participants (40; 70.2%) accessed a clinic for primary care. This is consistent with the number of such facilities in the area.

A bigger percentage of participants (20% or more) were not satisfied with the services that they received than what was found in international studies (Stillman et al. 2014, 2017). This might be because of the challenge of providing primary care of continuous high quality in developing countries (Onokerhoraye 2016).

### Access to primary care

Primary care was available to all participants. Most participants were happy with the number of staff at their healthcare facilities, an opinion that was confirmed by relatively short waiting times for both consultations and drugs. Other studies from Africa describe long waiting times (Munthali et al. 2019; Scheffler et al. 2015; Van Rooy et al. 2012; Vergunst et al. 2015) and insufficient numbers of staff (Scheffler et al. 2015; Van Rooy et al. 2012). The short waiting times are even more positive when one considers that not all facilities had an appointment system and even fewer gave appointments for a specific time. Not using appointments to schedule services has also been identified in Namibia and can lead to longer waiting times (Van Rooy et al. 2012).

The rather positive findings on staff attitude, preferential treatment and dignity are in contrast with most previous findings from Africa. Munthali et al. (2019) showed that persons with disabilities in Malawi experienced positive attitudes from service providers intermingled with discrimination, rudeness and prejudice. Other studies described discrimination (Bright & Kuper 2018; Munthali et al. 2019; Scheffler et al. 2015) against persons with disabilities, persons with disabilities being ridiculed (Mulumba et al. 2014) and abused (Baart & Taaka 2017; Mulumba et al. 2014) as well as negative attitudes and rudeness (Baart & Taaka 2017; Bright & Kuper 2018; Scheffler et al. 2015; Van Rooy et al. 2012).

A consultation time of less than 10 min is bothersome as it might be difficult to do a thorough physical assessment in such a short time in the light of the mobility challenges persons with SCI experience (Iezzoni 2006), especially since few of the hospitals had height-adjustable examination beds. Similarly to findings by Stillman et al. (2017) and Stillman et al. (2014), many participants were examined while sitting in their wheelchairs. Examining persons with SCI in their wheelchairs is unacceptable because important symptoms such as the initial stages of pressure ulcers, a serious and highly prevalent complication of SCI in developing countries (Zakrasek, Creasey & Crew 2015), might be missed. Baart and Taaka (2017) found in a systematic review in low- and middle-income countries that a lack of height-adjustable examination beds caused a challenge in providing healthcare services to persons with disabilities. Current study findings on the availability of equipment and that equipment were in working order are in contrast with many other African studies that found a lack of equipment and broken equipment (Bright & Kuper 2018; Eide et al. 2015; Vergunst et al. 2015). Not being able to access and use a bathroom is not acceptable. The lack of a call button is a serious safety oversight.

Participants thought that the staff’s knowledge on issues related to SCI was insufficient. Øderud (2014) identified similar challenges in neighbouring Zimbabwe. Services were mostly received from clinics and offered by general care providers who are not specifically trained in the management of SCI, a less common condition. Thus, persons with SCI might have to ‘educate’ their primary care providers in SCI-related issues as also suggested by Goodridge et al. (2015). This implies that persons with SCI must have a good understanding of their own condition.

The finding that availability of prescribed medication was overall generally poor could be because in Botswana all drugs are sourced from the central medical stores (CMSs). Therefore, if items are unavailable at the CMS, they would be unavailable to all facilities in the country. It appears that availability of prescribed medications was slightly better for health posts followed by clinics and worst in hospitals. This could possibly be attributed to prescribing patterns and hospitals being staffed with highly trained personnel as opposed to clinics and health posts where the training of staff might be of a more basic nature. Also, in most health posts and some clinics, the same person is prescribing and dispensing; it is likely that they might selectively prescribe medications, which they know are available. However, a lack of drugs and equipment has been identified as a barrier to healthcare access in other African settings (Eide et al. 2015; Mulumba et al. 2014; Vergunst et al. 2012, 2017) and the problem might be bigger than the supply system in Botswana.

Availability of SCI-related consumables was also generally poor, with hospitals faring slightly better in this regard. The reason for better availability of SCI-related consumables in hospitals might be attributable to the presence of more skilled personnel at hospitals as opposed to other facilities, and the finding that service providers at hospitals had more knowledge on SCI than at the other points of service provision. Doctors are authorised to order all items in the non-drug CMS catalogue and those not in the catalogue through special request procedures. In clinics, orders to CMS are performed by nurses and sometimes healthcare auxiliaries who are not authorised to order all CMS items and cannot put in special requests.

In addition to challenges with availability, accessibility challenges might have negatively impacted quality of care. The government of Botswana aims that every member of its population should be living within a 5 km radius of a primary care facility (Seitio-Kgokgwe et al. 2014). This aim was achieved for the study population. However, this distance seems to be too far for people with SCI and other physical disabilities as the majority of participants were not able to reach the facility with ease. Similar to participants in other studies (Vergunst et al. 2015), most of the participants used their wheelchairs to access primary care. However, as also found by Vergunst et al. (2015), some of them needed assistance to reach the facility with the wheelchair. This demonstrated a lack of independent community mobility and may be because of inappropriate mobility assistive devices (e.g. lack of motorised wheelchairs), a lack of access to public transportation and/or to the physical environment (Baart & Taaka 2017; Löfvenmark et al. 2016). Roads in Gaborone and surrounds are often not tarred and even the tarred ones do not have wheelchair-accessible walkways (Löfvenmark et al. 2016).

In the light of participants already struggling to access primary care, it is a challenge that referrals to other services were not supported by transport services as increased distances to these services will undoubtedly increase transport challenges and cost. Both variables have been identified as widespread and serious barriers to healthcare access in Africa (Baart & Taaka 2017; Bright & Kuper 2018; Eide et al. 2015; Øderud 2014; Van Rooy et al. 2012; Vergunst et al. 2017). The cost and effort involved might lead to nonattendance with detrimental health consequences.

## Limitations

All persons with SCI who could be identified in the study setting were asked to participate in the study. Even so some groups or individuals who differed in important ways from those identified might have been excluded such as persons who went to South Africa or elsewhere for care, those who were unable to access the system, for the same reasons as in the study but possibly extreme variations, and those who may have died prematurely. Therefore, one has to be careful when generalising findings to all persons with SCI in the study setting. It is especially important to take cognisance of the possibility that those who died of complications because of lack of access could influence the picture significantly. Not being able to include this group was an inherent and significant limitation of this study that affects all the results. Not being able to identify many participants with SCI because of non-traumatic causes was unfortunate as this subgroup might differ considerably from the larger population of persons with SCI and comparison between the two groups would have enhanced findings and recommendations from the study.

The questionnaire was self-developed and not tested for reliability and validity.

The primary author provided care to most of the participants in the past, although not at primary care facilities. This previous relationship might have influenced responses and caused social desirability bias in responses because participants might view him as part of the healthcare system and thus hesitate to share negative experiences.

## Conclusions

Primary care services were for the most part affordable and adequate. However, more than a third of participants indicated dissatisfaction with services. This in conjunction with accessibility, acceptability and availability challenges like transport and structural barriers, as well as a lack of knowledge, consumables and drugs, leads to the conclusion that there is room for improvement in the services.

### Recommendations

General awareness and knowledge on the management of SCI and the consumables and drugs persons with SCI need from primary care services must be raised. It is recommended that Spinalis, as the specialist SCI unit in the setting, develops and provides outreach training and ongoing support in this regard.

Primary care facilities should acquire at least one height-adjustable examining couch per facility and one toilet in each facility should adhere to international standards for wheelchair users.

Transport should be offered to people with mobility impairments who are referred to other services.

Local government representatives must be educated on the barrier that inaccessible road surfaces create in community mobility for persons using wheelchairs. Moreover, lobbies must promote the need for universally accessible public transport. Botswana Federation of the Disabled is ideally suited to take on this responsibility.

Botswana Ministry of Health should start a national data list on persons with SCI, because of both traumatic and non-traumatic causes.

It is recommended that the knowledge and understanding of people with SCI on their condition is studied. It is also recommended that the knowledge and understanding of primary level healthcare service providers on SCI is studied. Further study is also necessary to determine if RTCs do lead to unduly high incidence of SCI in Botswana. If this is indeed the case, health promotion activities focused on road safety should be initiated or enhanced in Botswana.
